# Testing an mHealth Momentary Assessment Routine Outcome Monitoring Application: A Focus on Restoration of Daily Life Positive Mood States

**DOI:** 10.1371/journal.pone.0115254

**Published:** 2014-12-16

**Authors:** Jim van Os, Philippe Delespaul, Daniela Barge, Roberto P. Bakker

**Affiliations:** 1 Department of Psychiatry and Psychology, Maastricht University Medical Centre, South Limburg Mental Health and Teaching Network, Maastricht, The Netherlands; 2 King’s College London, King’s Health Partners, Department of Psychosis Studies, Institute of Psychiatry, London, United Kingdom; 3 Leiden University Medical Centre, Leiden, The Netherlands; 4 Psychiatric Centre GGZ Centraal, Amersfoort, The Netherlands; Shanghai Mental Health Center, Shanghai Jiao Tong University School of Medicine, China

## Abstract

**Background:**

*Routine Outcome Monitoring* (ROM) is used as a means to enrich the process of treatment with feedback on patient outcomes, facilitating patient involvement and shared decision making. While traditional ROM measures focus on retrospective accounts of symptoms, novel mHealth technology makes it possible to collect real life, in-the-moment ambulatory data that allow for an ecologically valid assessment of personalized and contextualized emotional and behavioural adjustment in the flow daily life (mROM).

**Method:**

In a sample of 34 patients with major depressive disorder, treated with antidepressants, the combined effect of treatment and natural course was examined over a period of 18 weeks with Ecological Momentary Assessment (EMA). EMA consisted of repeated, within-subject, mini-measurements of experience (eg positive affect, negative affect, medication side effects) and context (eg stressors, situations, activities) at 10 unselected semi-random moments per day, for a period of six days, repeated three times over the 18-week period (baseline, week 6 and week 18).

**Results:**

EMA measures of emotional and behavioural adjustment were sensitive to the effects of treatment and natural course over the 18-week period, particularly EMA measures focussing on positive mood states and the ability to use natural rewards (impact of positive events on positive mood states), with standardized effect sizes of 0.4–0.5. EMA measures of activities, social interaction, stress-sensitivity and negative mood states were also sensitive to change over time.

**Conclusion:**

This study supports the use of mROM as a means to involve the patient in the process of needs assessment and treatment. EMA data are meaningful to the patient, as they reflect daily life circumstances. Assessment of treatment response with mROM data allows for an interpretation of the effect of treatment at the level of daily life emotional and social adjustment – as an index of health, obviating the need for an exclusive focus on traditional measures of ‘sickness’.

## Introduction

Routine Outcome Monitoring (ROM) in mental health services may be used to continuously update and direct clinical decision making with objective data. In addition, if the patient is involved in the data collection and interpretation, it will facilitate shared decision making. Finally, self-monitoring of symptoms, in combination with professional feedback [1], will help the patient gain insight in how symptoms vary as a function of treatment, and offer ways to cope with and manage symptoms. ROM activities thus may contribute to self-management of psychopathology [2].

ROM data may also secondarily be used, in aggregated form, as a local management information system yielding data on overall service performance. While potentially useful as a local management information tool, a well-known pitfall of ROM data collection is a shift away from enrichment of the clinical process to an exclusive focus on management information, as described in some countries. ROM should primarily be used to enhance clinical practice – its use as a management information tool is controversial, given the potential for bias, confounding and other case-mix issues that make it unsuitable for the purpose of comparative policy making.

ROM may be used across the range of mental disorders, and may be particularly instructive in the treatment of common mental disorders (anxiety and depression), in which treatment is expected to occasion improvement in the short to medium term but trajectories of change are highly personal and difficult to predict.

### ROM: focus on sick care or health care?

ROM activities typically are organized around standard self-report questionnaires. These, however, do not take into account that measures of psychopathology are both personalized as well as contextualized [3]–[6]. Personalized approaches aim to assess daily life moment-to-moment adaptation strategies: *when* does panic occur, *when* does an individual start worrying, develop paranoia, or loose cognitive capacity? Contrasting moments of good and failing adaptation (the daily life variability) discloses individual patterns of vulnerability and resilience and is invaluable to improve mental health outcomes on the basis of ROM. An mHealth approach to ROM (mROM) on the basis of ecological momentary assessment strives to measure prospective adaptation processes, offering personalized information that is useful for customizing interventions, and empowering the patient to become an active participant rather than a passive consumer. It provides a move away from ‘sick care’ based on symptoms towards ‘health care’ based on contextualized information in the daily life adaptation of the person to his environment. This increases transparency in the interventions and engages the patient more in his own treatment [7]–[9]. It may also be argued that evaluation of mental health care is more accurate if based on ecologically valid real life mROM outcome parameters rather than symptom-based measures embedded in traditional sick care ROM. For example, it has been shown that quality of life is driven by situational determinants that impact daily functioning and well-being in mental disorders. It is thus proposed that mROM approaches in mental health need to be developed in such a way that personalization and contextualization of psychopathology and treatment impact are made possible, in a collaborative and empowering fashion.

### mROM with Ecological Momentary Assessment Technology

**Figure 1:**
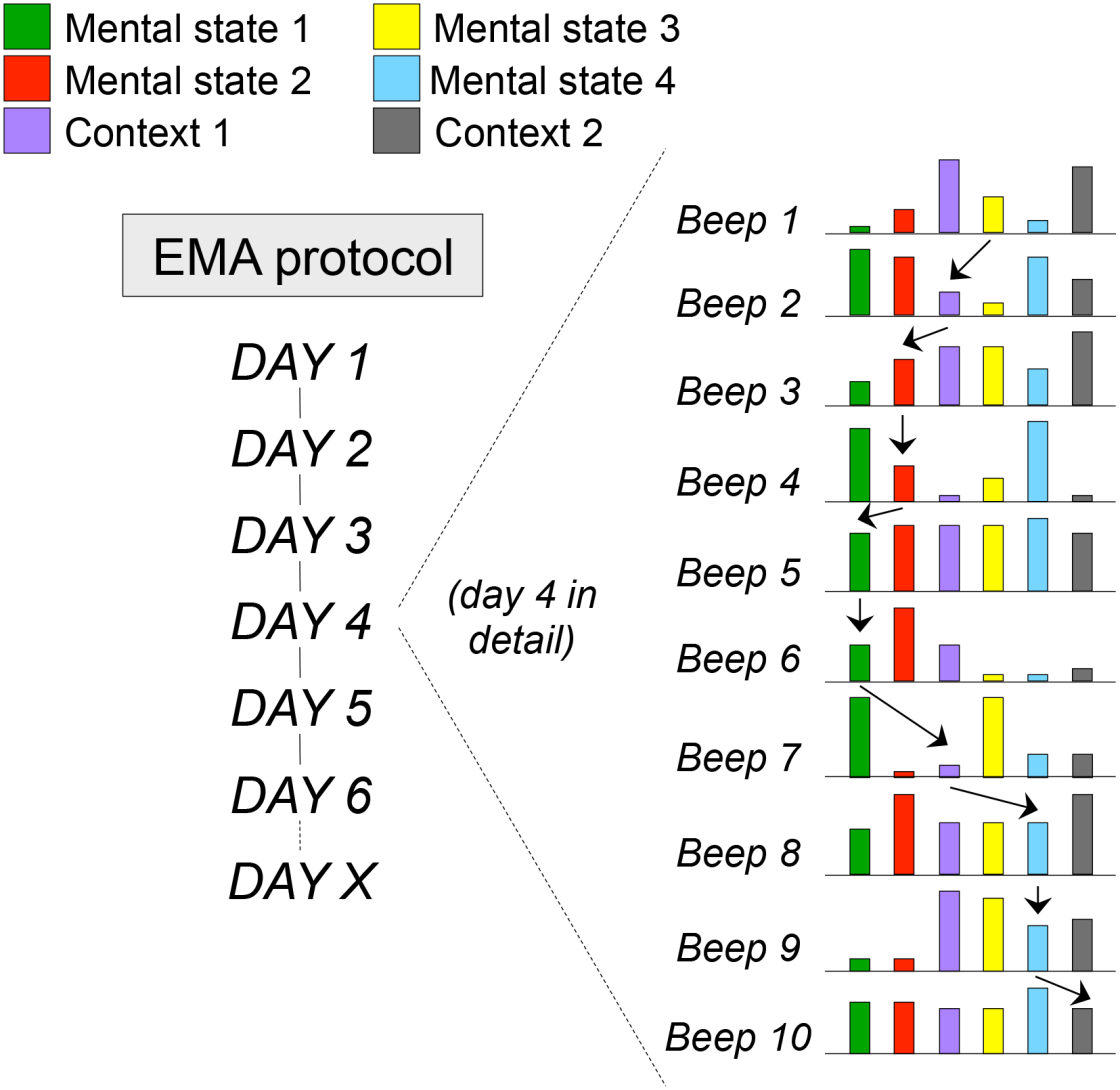
Principles of ecological momentary assessment, showing the details of a single day in the EMA paradigm. At 10 random moments during the day, mental states (eg anxiety, low mood, paranoia, being happy) and contexts (stress, company, activity, drug use) are assessed. The arrows represent examples of prospectively analysing the impact of mental states and contexts on each other over time, allowing for the construction of mROM measures such as stress-sensitivity (impact of stress on negative affect) or reward experience (impact of positive events on positive affect).

Personalization and contextualization of diagnosis and treatment in mental health care are possible with collaborative mHealth momentary assessment technology such as Ecological Momentary Assessment (EMA) or Experience Sampling Method (ESM) [3], [4]. Momentary assessment technology includes multiple repeated (within-subject) mini-measurements of experience (affect, wellbeing, motivation, salience, self-esteem, cognition, stress, anxiety, pain) and context (stressors, situations, activities, substance use, medication) at unselected semi-random moments in the flow of daily life (for example 8–10 times per day – Fig. 1).

Within-subject data provide patients and health professionals with the opportunity to follow intra-individual changes in relation to in-the-moment daily life situations and experiences. Momentary assessments reveal subtle repetitive and relevant patterns of experience in response to environmental and mental challenges that are not picked up by retrospective questionnaires or even interviews, and which the person is not consciously aware of. EMA allows the patient to have the ‘film’ rather than a ‘snapshot’ of daily life, uncovering important mechanisms and processes involved in the assessment and treatment of mental disorders [3], [4]. As it assesses the occurrence of mental states and contexts in the flow of daily life, EMA can accurately follow the impact of mental states and context on each other over time (arrows Fig. 1), which can be fed back to the patient, thus making explicit previously implicit dysfunctional patterns of emotions and reactivity.

### Evidence that mHealth-EMA technology is suitable for mROM disease management of mental problems including self-diagnosis, self-monitoring and self-management

An extensive body of work employing ambulatory momentary assessment technology, using EMA, has shown its usefulness for clinical practice, and its potential for diagnosis in psychiatry, including prediction of personal needs [10], [11]. EMA measures thus have been shown to underlie and mediate many personal clinical measures including symptoms, prognosis and treatment response (Table 1). In addition, there is evidence that use of add-on mHealth assessment and feedback is therapeutic, improving outcomes of patients, empowerment of patients and cost-effectiveness of treatment (Table 1). While this work only represents the beginning of the exploration of mHealth technology in health care, it suggests that it is well suited to aid efforts to improve diagnosis and treatment in the area of mental and somatic health. In addition, recent work suggests that patients who engage in EMA and receive systematic feedback on their pattern of affective responses to the environment, helping them to access and alter implicit behavioural and emotional repertoires, benefit therapeutically, over and above traditional treatments [12]. Thus mHealth based on EMA may be useful for mROM and diagnosis, monitoring of psychopathology in response to treatment and for interventions [3]–[5], [13], [14]. EMA mHealth interventions are not only suitable as stand-alone self-management, but may be particularly useful also as add-on to existing 1-on-1 pharmacological and non-pharmacological treatments, making them more effective or reducing the amount of therapy contact [12].

**Table 1:** Selected mHealth findings based on Ecological Momentary Assessment.

MECHANISM	EVIDENCE (PA = positive affect; NA = negative affect)
DISEASE MANAGEMENT: PERSONAL DIAGNOSIS SYMPTOMS AND NEEDS	Patients suffering from psychosis hallucinate in highly personal patterns [47]
	Anxiety is an important precursor factor for hallucinations [48].
	Self-esteem boosting interventions administered in the first 6 weeks after admission to healthcare services improve the subsequent course of negative symptoms in SMI patients [49]
	Identification of ‘comfort zones for patients in certain social environments [50]
	Personal variation in depression contributes to the formation of suicidal ideation [51]–[53]
	Formation of clinically detectable mental symptoms is induced by emotion-dependent alterations in the level of transfer of momentary experience of aberrant salience [54].
	Onset of psychotic symptoms is mediated in part by the tendency to develop momentary psychotic responses after momentary increases in negative affect [55]
	Paranoid delusionality is driven by momentary negative emotions and reductions in momentary self-esteem [56], in interaction with momentary stress in daily life [57]
	Onset of depressive symptoms is predicted by baseline stress sensitivity (momentary negative affective response to momentary stressful events) in the EMA paradigm [58]
	Individuals who placed greater emphasis on controlling their thoughts had greater variability in their self-esteem during the stress condition, which predicted psychopathology severity [59]
	The construct of negative schizotypy is associated with underlying momentary mental states and ecological interactions in the EMA paradigm, including decreased momentary positive affect and pleasure in daily life, and decreases in momentary social contact and interest [60]
	The construct of negative symptoms in serious mental illness does not translate to altered emotional processing in the EMA paradigm: Lower rather than higher levels of negative symptoms predicted an abnormal pattern of emotional processing in daily life [61]
	Specific mHealth momentary-level interactions between symptoms, and between symptoms and environment, sensitively predict treatment needs over and above traditional diagnosis [6]
	Specific mHealth measures of symptom-environment interactions predict personal rehabilitation needs in patients with serious mental illness [3], [10]
DISEASE MANAGEMENT: INSIGHT PERSONAL VULNERABILITY AND RESILIENCE	Exposure to early trauma increases sensitivity to stress in daily life, both in terms of emotional response (momentary negative affective response to momentary stressful events in daily life) and aberrant salience response (momentary psychotic response to momentary stress in daily life) [62] [63], [64]. The path from early adversity to psychopathology is potentiated by depression liability, impacting on dysfunctional emotional processing of anomalous experiences [65].
	Growing up in an urban environment does not predict altered sensitivity to daily stress [66]
	The psychopathological effects of cannabis are mediated by induction of momentary experiences of aberrant salience in the flow of daily life [67]
	The influence of major Life Events (LE) on onset of serious mental illness is mediated by the cumulative impact of LE on momentary stress sensitivity in the EMA paradigm [68].
	Momentary positive emotions in the EMA paradigm attenuate genetic effects on negative mood bias in daily life [33].
	Vulnerability for serious mental illness is associated, at EMA level, to both the momentary psychosis-inducing and the momentary mood-enhancing effects of cannabis [67], [69].
	Individuals at high risk of psychopathology are more sensitive to everyday stressors [70]
	Risk for mental disorders is mediated by enhanced momentary aberrant salience and momentary negative affect in response to stress in the flow of daily life [40], [71]–[73]
	Familial risk for mental disorder is expressed as a greater level of momentary transfer (or persistence) of experience of aberrant salience in the flow of daily life [54]
	Individuals at risk for depression and psychotic disorder have a different diurnal cortisol profile than those without, suggesting that altered hypothalamic-pituitary-adrenal axis functioning in response to stress is an indicator of susceptibility to depression and psychosis [74], [75]
	Borderline patients react stronger than patients with psychosis and healthy controls to small disturbances that happen in the natural flow of daily life. Altered negative affective and aberrant salience stress reactivity may define borderline personality disorder [76], [77]
	Neuroticism as measured by Eysenck questionnaire may index an environmental risk for decreased daily life levels of PA, and a genetic risk for increased NA variability [78]
DISEASE MANAGEMENT: PERSONAL MONITORING TREATMENT RESPONSE	In depression, baseline Reward Experience (momentary positive affective response to positive events) and baseline negative affect variability (variability in momentary negative affective response to negative events) in the EMA paradigm accurately predict outcome [37].
	In antipsychotics with tight binding to the dopamine D2 receptor, increased levels of estimated D(2) receptor occupancy is associated with decreased feelings of momentary positive affect (PA) and increased feelings of negative affect (NA) in the EMA paradigm [79]
	In depression, early change in positive rather than negative emotions in the EMA paradigm best predicted response to treatment, over and above changes in traditional rating scales [80]
	In depression, future response to treatment was associated with altered baseline dynamics between NA and PA in the EMA paradigm: daily life boosts of PA were followed by a stronger suppression of NA over subsequent hours than in other depressed groups or controls [81]
	Remission criteria for schizophrenia are manifested in daily life as fewer instances of momentary aberrant salience, better momentary mood states and partial recovery of momentary reward experience [82]
	Depression treatment with Mindfulness-based Cognitive Therapy is mediated by increased experience of momentary positive emotions as well as greater appreciation of, and enhanced responsiveness to, pleasant daily-life activities in the EMA paradigm [35]
	Response to antidepressant medication is mediated by increase in Reward Experience (momentary positive affective response to positive events) rather than reduction in Stress-Sensitivity (momentary negative affective response to negative events)[16]
	The therapeutic effect of physical activity on mood is mediated by momentary increases in positive affect rather than reduction in negative affect in the EMA paradigm [83]
mHealth DISEASE MANAGEMENT & COST-EFFECTIVENESS	Add-on mHealth self-management and feedback aimed at increasing resilience and use of natural rewards produced greater improvement in depressed patients on antidepressants [12].
	Add-on mHealth self-management and feedback in depression resulted in increased experience of empowerment [84].
	Add-on mHealth self-management and feedback in depression is cost-effective [85]

Whilst these results are encouraging, it should be noted that most of the work was carried out with simple pencil-and-paper technology, and therefore is not suitable for large-scale introduction in health care. Recently, stand-alone electronic devices have been introduced which can be used to conduct EMA (www.psymate.eu) however the real challenge for the dissemination in clinical practice is to develop a cloud-based technology that can harvest data collected by mobile phones and process the results to enhance the therapeutic work in clinical decision making and instruction.

### Study aims

In the current study, we wished to test to whether EMA assessments may be useful as an mROM tool, i.e. to what degree they are sensitive to change over time in ecologically valid outcomes in a group of patients treated for depression and followed for a period of 18 weeks. The focus was on change (reflecting natural course and treatment effect) in the eight main EMA measures of negative affect, positive affect, stress reactivity, reward function, activity level, social interaction and treatment side effects. These measures were chosen given their transdiagnostic [15] value as independent EMA indicators of emotional and behavioural adjustment in daily life (Table 1) that are relatively independent of each other. For example, measures of momentary positive affect are only weakly related to momentary measures of negative affect [16]. Similarly, reduced ability to experience positive mood in response to positive environmental stimuli and the tendency to experience negative mood in response to negative environmental stimuli are transdiagnostic features of mental illness that are only weakly associated with each other [17]. In addition, the proposed mROM EMA-measures are associated with treatment needs, over and above traditional measures of psychopathology [6].

In order to examine these issues, we carried out an observational analysis in a subgroup of subjects in the treatment arm of a randomised controlled trial, the result of which were published before [18]–[20] and are not related to the current report.

## Methods

### Subjects

Eighty-three patients with a DSM-IV diagnosis of current major depressive disorder (MDD) were recruited in eight primary care practices in the Netherlands (for full details concerning diagnosis and screening, see Barge-Schaapveld et al., 2002 [18]). Data were collected in the period between 1995 and 1996. Inclusion criteria were: age between 18 and 65 years, a score at study entry of ≥18 on the 17-item Hamilton Depression Rating Scale (HDRS), and a score ≥4 on the Clinical Global Impression (CGI)[21]. Exclusion criteria included major medical disorders and current use of psychotropic medications, except for the occasional use of temazepam.

Subjects gave written informed consent. The study was approved by the standing medical ethics committee. Ethics approval in the Netherlands routinely includes: refusal to take part cannot impact treatment in any way, the right to consult an independent clinician, the right to discontinue at any moment without having to provide a reason.

All analyses in this report refer to the subgroup of patients in the treatment arm of the RCT, who were treated with an antidepressant, in order to model the situation of outcome monitoring (monitoring the effect of treatment and natural course) in routine clinical practice. As placebo is not a recognised active treatment in routine clinical practice, the placebo arm of the RCT was not included in the analysis.

### Study design

The study was conducted in a primary care setting, in a sample of outpatients with MDD. During an initial baseline week, participants received no treatment in any form. Thereafter, patients were randomly assigned to twice-daily, double-blind, six-week treatment with either a tricyclic antidepressant (imipramine: starting dose of 50 mg/day, increased to 200 mg/day over the first week of treatment) or placebo (starting with one capsule per day, increased to 4 capsules over the first week of treatment). In case of intolerance, the dose could be decreased to either 100 mg/day of imipramine or 2 placebo capsules per day. All subjects participated in the EMA procedure at baseline and at week 6. Patients were offered the possibility to again participate in EMA at 18 weeks.

### Ecological Momentary Assessment

EMA is a structured experience sampling diary technique to assess subjects in their daily living environment, which has been validated for the use of studying the immediate effects of stressors on mood [3], [22], [23]. Subjects received a digital wristwatch and a set of EMA self-assessment forms collated in a booklet for each day. The wristwatch was programmed to emit a signal (“beep”) at an unpredictable moment in each of ten 90-minute time blocks between 7∶30 and 22∶30, on six consecutive days. After each beep, subjects were asked to fill out the EMA self-assessment forms to record current thoughts, current context (activity, persons present, and location), appraisals of the current situation, and mood. All self-assessments were rated on 7-point Likert scales. Trained research nurses explained the EMA procedure to the participants during an initial briefing session. Subjects were instructed to complete their reports immediately after the beep, thus minimizing memory distortion, and to record the time at which they completed the form. To determine whether subjects completed the form on time, self-reported times were compared to the actual beep times. All reports not filled in within 15 min after the beep were excluded from the analysis, since previous work has shown that reports completed after this interval are less reliable and consequently less valid. For the same reason, subjects with fewer than 20 valid reports (out of 60 maximum) were excluded from the analysis.

In order to test EMA test-retest reliability, a group of 22 healthy individuals, with socio-demographic characteristics similar to those of the patient group, were examined in order to provide normal reference values for EMA measures. In this group, mean levels of PA, NA and appraisal of activities were stable over a period of six weeks [24].

### EMA predictors of treatment outcome at 6 and 18 weeks

#### Mood states: NA and PA

At each beep, EMA mood adjectives were assessed, conform previous work [18]. Factor analysis, using principal component analysis with oblique rotation, was used to generate a factor representing positive affect (PA) and one representing negative affect (NA). The mood adjectives ‘cheerful’, ‘satisfied’, ‘energetic’, ‘calm’, ‘alert’ ‘enthusiastic’, ‘strong’ and ‘happy’ loaded on the PA factor while ‘hostile’, ‘depressed’, ‘tensed’, ‘insecure’, ‘lonely’, ‘anxious’, ‘guilty’, ‘hurried’ and ‘irritable’ loaded on the NA factor. The item ‘I feel tired’ was left out of the constructed scales, because its loading was not specific for either NA or PA.

#### Appraisals of activity and social interaction

In order to define stress and reward in relation to ecologically valid daily activities, consistent with previous work in this sample, EMA self-rated appraisals of ongoing activities were used. Self-report items on current activity were rated on a Likert scale (with 1 =  not at all and 7 =  very). Factor analysis supported inclusion of three items on activity appraisal, namely: ‘do you enjoy this activity, ‘does this activity require effort’, ‘is this a challenge’ and ‘are you skilled at doing this activity’. Based on these ratings, variables reflecting ‘A*ctivity Stress*’ and ‘*Activity Reward*’ were created. For *Activity Stress*, the positive appraisals ‘do you enjoy the activity’ and ‘are you skilled at doing this activity’ were first recoded in reverse, so that higher scores represented more negative appraisals (low enjoyment and low skill, respectively), Then, low scores (≤4) on all four scales were set to zero and the remaining values recoded (5 = 1, 6 = 2, 7 = 3), so that only appraisals with a negative valence contributed to the summed *Activity Stress* score. Similarly, to create the variable *Activity Reward*, the item ‘does this activity require effort’ and ‘is this a challenge’ was first recoded in reverse so that high scores reflected lower appraised effort and less of a challenge. Subsequently, low scores (≤4) on these recoded items and the other two appraisal items were set to zero, and higher scores were recoded (5 = 1, 6 = 2, 7 = 3) before calculating a sum score for *Activity Reward.*

‘*Stress-Sensitivity*’ was conceptualized in the analyses as the effect of stressful daily life activities on NA (i.e. the effect of the *Activity-Stress* score on NA). Similarly, ‘*Reward Experience*’ was conceptualized as the effect of positively appraised daily life activities on PA (i.e. the effect of the *Activity Reward* score on PA). In addition, ‘*Reward Persistence*’ was conceptualized as the effect of positively appraised daily life activities at the previous beep on PA at the subsequent beep, controlling for PA at the previous beep (i.e. the effect of the lagged *Activity Reward* score on PA, controlling for lagged PA).

EMA appraisal of social activity was scored if the participant indicated he was with at least on other person at the moment of the beep (more than 60% of beeps), at which moment the participant was asked to rate to what degree (i) he would rather be alone (hereafter: Social Discomfort) and (ii) he was doing something together with this person or persons (hereafter: Social Activity).

Medication side effects were assessed within the EMA paradigm at each beep, also scored on 7-point Likert scales, assessing headache, drowsiness, nausea, dry mouth, dizziness and ‘other complaint’. The total score of these six items was the *Side Effect Score*.

### Statistical analyses

EMA data have a hierarchical structure. Thus, multiple observations (level 1) are clustered within subjects (level 2). Multilevel analysis takes the variability associated with each level of nesting into account [25]. Multilevel linear regression analyses, using the XTREG command in Stata 13.0, were applied to the data, analysing continuous outcomes. As participants participated in multiple periods of EMA (baseline, week 6 and week 18) a dummy was included for time. Standardized beta coefficients (ß) are reported.

Analyses assessed the main effect of time (1 = baseline, 2 = week 6 and 3 = week 18 – independent variable, representing the combined effect of treatment and natural course) on NA, PA, *Social Discomfort*, *Social Activity* and *Side Effect Score* (dependent variables). In addition, in order to analyse the effect of time on *Stress Sensitivity* and *Reward Experience/Persistence*, interactions were fitted between time and *Activity- Stress* in the model of NA, and between time and (lagged) *Activity Reward* in the model of PA, adjusting for lagged PA in the model with lagged *Activity Reward*. Effect sizes of the interactions between time on the one hand and EMA predictors of outcome on the other were calculated using the Stata Margins command.

#### Sensitivity analyses

In order to examine to what degree EMA measures of ROM are sensitive to the number of days patients report experiences, or the number of beeps per day, sensitivity analyses were carried out, restricting the EMA paradigm to 4 days (excluding the last two days, 10 beeps per day), 8 beeps (6 days, excluding the last two beeps) and 8 beeps per day for 4 days.

## Results

### Patient sample

Of 83 patients with MDD, 11 were excluded on the basis of the described inclusion and exclusion criteria. Reasons were use of psychotropic medication, abnormal ECG and HDRS lower than 18; one eligible subject was excluded due to an error in the randomization procedure. Five subjects were later excluded, because they failed to complete at least 20 valid EMA reports at baseline, (see *Ecological Momentary Assessment*, above), leaving 66 patients, of whom 34 were in the imipramine group. At week 6, 23 patients in the imipramine group had valid EMA data. At week 18, 17 patients in the imipramine group presented with valid EMA data. The main reason for drop out at both week 6 and week 18 was fatigue with the pen-and-paper EMA procedure.

In the 34 patients in the imipramine group at baseline, attrition at week 6 or week 18 was not associated at baseline with PA, NA, *Activity Stress, Activity Reward, Side Effect Score, Social Activity and Social Discomfort*, with the exception of an association between attrition at week 18 and baseline *Social Discomfort* (p = 0.041) indicating greater levels of discomfort in those who dropped out at week 18.

### Subject characteristics

At baseline, patients ranged in age from 25 to 59 years (mean = 42.7). The majority were women (72%) and married (68%). Most participants had a regular job (46%) or were housewives (25%).

In the 34 patients treated with imipramine, baseline average HDRS score was 24.4 (SD = 3.8; range: 19–34). At week 6, average HDRS score was 8.5 (SD = 6.3; range: 0–24), and 5.6 at week 18 (SD = 7.4; range: 0–23).

### Effect of time on mROM measures

There were significant main effects of time on NA, PA, *Social Discomfort*, *Social Activity*, *Side Effect Score*, *Stress Sensitivity*, *Reward Experience and Reward Persistence*, both at week 6 and week 18 (Table 2).

**Table 2:** Effect of time (baseline, week 6, week 18) on mROM measures of NA, PA, *Social Discomfort*, *Social Activity* and *Side Effect Score, Stress Sensitivity*, *Reward Experience and Reward Persistence* in 34 patients treated for depression with an antidepressant.

	STANDARD PROTOCOL				SENSITIVITY ANALYSES											
	EMA 6 days, 10 beeps per day				EMA 4 days, 10 beeps per day				EMA 6 days, 8 beeps per day				EMA 4 days, 8 beeps per day			
	Week 6		Week 18		Week 6		Week 18		Week 6		Week 18		Week 6		Week 18	
mROM measures	Beta	P	Beta	p	Beta	p	Beta	*P*	Beta	p	Beta	*p*	Beta	p	Beta	*p*
Negative affect	−0.19	<0.001	−0.26	<0.001	−0.19	<0.001	−0.26	<0.001	−0.20	<0.001	−0.27	<0.001	−0.21	<0.001	−0.27	<0.001
Positive affect	0.26	<0.001	0.47	<0.001	0.24	<0.001	0.44	<0.001	0.26	<0.001	0.48	<0.001	0.25	<0.001	0.45	<0.001
Reward Experience*	0.21	<0.001	0.40	<0.001	0.19	<0.001	0.43	<0.001	0.22	<0.001	0.36	<0.001	0.21	<0.001	0.37	<0.001
Reward Persistence**	0.20	<0.001	0.37	<0.001	0.18	<0.001	0.38	<0.001	0.22	<0.001	0.33	<0.001	0.20	<0.001	0.32	<0.001
Social Discomfort	−0.10	<0.001	−0.18	<0.001	−0.08	<0.001	−0.20	<0.001	−0.09	<0.001	−0.18	<0.001	−0.06	0.021	−0.20	<0.001
Social Activity	0.09	<0.001	0.09	<0.001	0.12	<0.001	0.11	<0.001	0.09	<0.001	0.10	<0.001	0.12	<0.001	0.13	<0.001
Stress Sensitivity#	−0.07	0.001	−0.05	0.004	−0.11	<0.001	−0.05	0.020	−0.08	<0.001	−0.05	0.006	−0.11	<0.001	−0.05	0.047
Side Effect Score	0.04	0.004	−0.04	0.003	0.07	<0.001	−0.04	0.028	0.02	0.118	−0.06	<0.001	0.06	0.005	−0.05	0.015

*Represents change in the effect of *Activity Reward* on PA between from baseline to week 6, and from baseline to week 18 (assessed as the interactions between time and *Activity Reward* in the model of PA).

**Represents change in the effect of lagged *Activity Reward* on PA between from baseline to week 6, and from baseline to week 18 (assessed as the interaction between time and lagged *Activity Reward* in the model of PA, adjusting for lagged PA).

# Represents change in the effect of *Activity Stress* on NA from baseline to week 6, and from baseline to week 18 (assessed as the interaction between time and *Activity Stress* in the model of NA).

The largest effect sizes, with the clearest dose-response over time, were for the positive affect-related measures (*PA, Reward Experience and Reward Persistence*) with effect sizes of 0.4–0.5 at week 18. Effect sizes were smaller for the other measures, but mostly highly significant.

Sensitivity analyses indicated that results were not impacted by reducing EMA sampling days to 4, and/or EMA sampling moments to 8 (Table 2).

## Discussion

### Findings

This study showed that ecological momentary assessment data, collected in the flow of daily life, and reflecting daily life emotional and social adjustment, are sensitive to the management of depression in mental health settings. Measures reflecting positive adjustment, positive affect and the ability to generate positive affect in response to natural rewards had greatest effect sizes. This suggests that the process of ‘getting better’ in disorders of emotional dysregulation may be about restoration of positive mood, in addition to, or possibly over and above, reducing negative mood [26].

### mROM: a focus on positive mood states?

The focus on restoring positive mood states in patients is not adequately addressed by available routine outcome strategies. Routine outcome measurement has a focus on symptoms, in the context of ‘*sick care*’ rather than resilience, in the context of ‘*health care*’. Indeed, current diagnostic systems do not include decreased positive mood as an identifying feature for any disorder. There is strong evidence, however, that the onset of depressive disorder is associated with loss of positive mood states [27]–[29], particularly in the context of poor sleep [30], as well as with the course and outcome of depression [31], [32]. Similarly, the ability to make use of natural rewards (mediated by positive affect) protects against the risk-increasing effects of environmental and genetic risk factors [33], [34], may mediate the effect of pharmacotherapy and psychotherapy in the treatment of depression [16], [35] and predict treatment response [36]–[38]. In addition, a recent randomised controlled trial in MDD patients treated with antidepressants showed that standardized feedback of EMA-based measures of positive mood and ability to make use of natural rewards resulted in enhanced patient outcomes.

Routine outcome monitoring with a focus on positive mood states and the ability to use natural rewards is in line with recent theories about the role of positive mood in facilitating the development of a range of physical, cognitive (coping), and social (interpersonal trust) resources, protecting against negative mood states [39].

The importance of positive mood states is transdiagnostic, extending to psychotic disorder [40], [41], anxiety disorders [42] and addiction [43]. The mROM measures of daily life emotional and social adaptation, as proposed in this article in the context of the treatment of common mental disorder, may therefore be suitable across different diagnostic categories, simplifying its use in mental health settings, and enhancing cross-diagnostic comparisons of outcomes. mROM may be used as a complement to traditional symptom-based Rom strategies.

### Development of digital and automated mROM systems based on EMA

**Figure 2:**
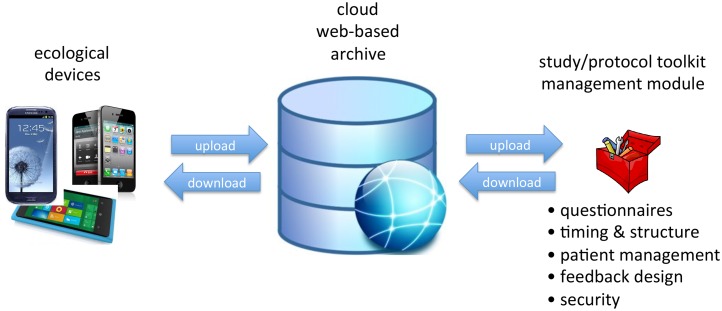
Principles of mROM.

The principle of mROM using EMA can be automated as depicted in Fig. 2, making it much easier for patients to use in clinical practice, increasing participation [8], [9]. The automated mROM ecological momentary assessment solution consists of: a ***device*** (eg iOS and Android smartphones), a special purpose ***application*** (*app*) that will be used by clinicians and researchers with specific ‘pro’ functionality; a ***communication protocol*** that manages the cloud/database and uploads protocols to registered users who will collect ecological data of environment-related mental and behavioural reactivity, customized for different populations and targets; a ***toolkit that manages protocols*** (sets of questions that will be assessed under specific pre-defined circumstances, e.g. random within 90 minute blocks; increasingly frequent during periods of anxiety); a ***module that manages subject registration*** within a study/protocol-combination; a toolkit that customizes and ***manages feedback*** (feedback can be static [predefined reports] or interactive [responsive to queries]; ***a cloud (distributed) host*** facility with adequate security to contain mental health data, managed data integrity and backup.

Given the high level of patient involvement in the data collection, use of mROM based on EMA has the advantage that it protects against the tendency for ROM data collection to be used exclusively as a management information tool. Patients naturally expect the EMA data they collected in daily life to be used in the process of diagnosis and treatment, making EMA a natural inductor of shared decision making. Clinicians require clinical feedback reflecting in-the-moment real life circumstances rather than retrospective interpretations that may be contaminated in many ways. A combination of mROM and traditional retrospective questionnaire data allows for a more refined interpretation of the effects of treatment and time in individual patients.

### Methodological issues

The results should be viewed in the light of several methodological issues. First, EMA measurements are based on subjective reports. Therefore, it can be argued that the results are not psychometrically precise. However, although subjective reports are considered less reliable (e.g. do all subjects interpret or answer the questions identically?), previous research indicates that subjective reports can be valid, and that the validity of objective reports should not be taken for granted [44]. Second, EMA is a daily life assessment technique in which subjects have to comply with a paper-and pencil diary protocol without the researcher being present. However, some authors have cast doubt on the reliability and subject compliance in paper-and pencil EMA studies, favouring the use of electronic devices [45]. In a comparative study, Green et al. concluded that both methods yielded similar results. In addition, a study using a signal-contingent random time sampling procedure with multiple observations per day, similar to the protocol used in the current study, found evidence that underscores the validity of the paper-and-pencil random time self-report data in the current study [46]. Third, the study was not conducted specifically for the purpose of routine outcome monitoring in clinical practice. That is, data collected by the patient were not used to feed the cycle of treatment, response, feedback and further treatment in routine clinical practice. Previous EMA studies, however, have demonstrated that feedback of EMA data in clinical practice results in enhanced treatment results, particularly if focussed on measures of positive affectivity in the context of activities and social interaction, similar to the measures used in this study [12]. Fourth, attrition was substantial, particularly from week 6 to week 18, introducing the potential for selection and weakening statistical power. More work is required in larger samples to validate these results. Finally, this study focussed on depression, and although we propose that the mROM measures used in this report are of transdiagnostic relevance more work is required to validate this method outside the area of common mental disorder, such as psychosis and addiction.

### Conclusion

This study supports the use of mROM as a means to involve the patient in the process of treatment. EMA data are meaningful to the patient, as they reflect daily life circumstances. Assessment of treatment response with mROM data thus allows for an interpretation of the effect of treatment at the level of daily life emotional and social adjustment – where it counts, obviating the need for an exclusive focus on measures of ‘sickness’.
